# Toxicity Overrides Morphology on *Cylindrospermopsis raciborskii* Grazing Resistance to the Calanoid Copepod *Eudiaptomus gracilis*

**DOI:** 10.1007/s00248-016-0734-8

**Published:** 2016-02-18

**Authors:** Luciana M. Rangel, Kemal A. Ger, Lúcia H. S. Silva, Maria Carolina S. Soares, Elisabeth J. Faassen, Miquel Lürling

**Affiliations:** Departamento de Botânica - Museu Nacional, Universidade Federal do Rio de Janeiro, Rio de Janeiro, 20940-040 Brazil; Department of Environmental Sciences, Aquatic Ecology and Water Quality Management Group, Wageningen University, Wageningen, The Netherlands; Laboratório de Ecofisiologia e Toxicologia de Cianobactérias, Instituto de Biofísica Carlos Chagas Filho, Universidade Federal do Rio de Janeiro, Avenida Carlos Chagas Filho, CCS—Bloco G—Cidade Universitária, Rio de Janeiro, 21941599 RJ Brazil; Departamento de Ecologia, Universidade Federal do Rio Grande do Norte, Natal, RN Brazil; Departamento de Engenharia Sanitária e Ambiental, Universidade Federal de Juiz de Fora, Juiz de Fora, MG Brazil; Department of Aquatic Ecology, Netherlands Institute of Ecology (NIOO-KNAW), Wageningen, The Netherlands

**Keywords:** Cyanobacteria, Feeding inhibition, Harmful algal blooms, Saxitoxins, Temperature, Zooplankton

## Abstract

Toxicity and morphology may function as defense mechanisms of bloom-forming cyanobacteria against zooplankton grazing. Yet, the relative importance of each of these factors and their plasticity remains poorly known. We tested the effects of chemical and morphological traits of the bloom-forming cyanobacterium *Cylindrospermopsis raciborskii* on the feeding response of the selective feeder *Eudiaptomus gracilis* (Calanoida, Copepoda), using a saxitoxin-producing strain (STX+) and a non-saxitoxin (STX−)-producing strain as food. From these two chemotypes, we established cultures of three different morphotypes that differed in filament length (short, medium, and long) by incubating the strains at 17, 25, and 32 °C. We hypothesized that the inhibitory effects of saxitoxins determine the avoidance of *C. raciborskii*, and that morphology would only become relevant in the absence of saxitoxins. Temperature affected two traits: higher temperature resulted in significantly shorter filaments in both strains and led to much higher toxin contents in the STX+ strain (1.7 μg eq STX L^−1^ at 17 °C, 7.9 μg eq STX L^−1^ at 25 °C, and 25.1 μg eq STX L^−1^ at 32 °C). Copepods strongly reduced the ingestion of the STX+ strain in comparison with STX− cultures, regardless of filament length. Conversely, consumption of shorter filaments was significantly higher in the STX− strain. The great plasticity of morphological and chemical traits of *C. raciborskii* and their resultant contrasting effects on the feeding behavior of zooplankton might explain the success of this cyanobacterium in a variety of aquatic environments.

**Electronic supplementary material:**

The online version of this article (doi:10.1007/s00248-016-0734-8) contains supplementary material, which is available to authorized users.

## Introduction

Bloom-forming cyanobacteria dominate the phytoplankton community in eutrophic waters [1], partly due to the wide range of plastic traits [2] that enable them to efficiently compete for resources. Although successful resource competition may explain potential population growth, the formation of blooms can occur when grazing pressure on the bloom-forming organisms is kept at a sufficiently low level [3]. Thus, in addition to efficient resource use, adaptations to grazer pressure are major cyanobacterial traits that regulate the abundance of bloom-forming species in eutrophic environments [4].

Harmful cyanobacteria possess two major traits that limit zooplankton grazing pressure: (i) morphological features such as large unit size or mucous layers interfering with ingestion and digestion [5] and (ii) production of metabolites with feeding inhibitory or toxic activity [6–8]. Toxicity and morphology are the two major food quality properties of cyanobacteria that constrain zooplankton grazing [9]. Yet, the relative role of each of these traits on widespread zooplankton feeding behavior is still unknown.

Cyanobacterial proliferation commonly shifts zooplankton communities to smaller and more tolerant grazers [10, 11]. Zooplankton organisms that survive during blooms do so either by an improved physiological resistance to ingested cyanobacterial metabolites or by avoiding the ingestion of potentially toxic cyanobacteria via selective grazing [12]. Many copepods are able to reach high biomass in systems dominated by cyanobacteria [13, 14]. In general, copepods are highly selective in their feeding and can discriminate prey by size, taste, and toxicity [15–17]. Yet, most of the work on zooplankton–cyanobacteria interactions has focused on large generalist grazers because of their potential to control blooms [9]. Selective grazers often replace large generalists when cyanobacteria are dominant, therefore their role on bloom dynamics merits further attention [4].

*Cylindrospermopsis raciborskii* (Woloszyńska) Seenayya et Subba Raju is one of the most widespread bloom-forming cyanobacteria in freshwater systems due to its recent expansion toward temperate regions [18]. This spread has been linked to its high tolerance for a wide range of climatic conditions [19, 20]. *C. raciborskii* is a filamentous species which can produce toxins such as cylindrospermopsins and paralytic shellfish toxins [21]. Some environmental factors such as light, temperature, and nutrients are known to affect the toxin production and morphology of *C. raciborskii* [22–24]. Nevertheless, our understanding of the plasticity of these two traits is incipient, as well as its effects on zooplankton. Given that both filament size and toxicity likely act as grazing avoidance mechanisms, distinguishing between their relative roles is a critical step toward understanding bloom dynamics. A recent study indicated that copepods might use toxicity-related signals (i.e., cylindrospermopsin) to avoid ingestion of toxic *C. raciborskii* [25]. However, it is still not understood if this pattern is in fact related to this toxin and how differences in filament length affect the feeding habits of zooplankton. Accordingly, this study aims to determine the role of cyanobacterial toxicity and morphology on zooplankton feeding responses, using a saxitoxin-producing (STX+) and a non-saxitoxin (STX−)-producing *C. raciborskii* strain as food for the calanoid copepod *Eudiaptomus gracilis*, a common selective grazer in Eurasia [12]. The reported optimum range of prey size for diaptomids is between 10 and 50 μm [26]. From each of the two chemotypes, we established three morphotypes of differing filament length by incubating the strains at different temperatures (17, 25, and 32 °C). We expected temperature to affect the morphology of the two strains as well as the toxicity of the saxitoxin-producing strain. We hypothesized that toxicity would be the dominant trait to reduce grazing, and that filament size would only play a role in the absence of toxicity. Thus, we expected that (i) the saxitoxin-producing strain would be consumed less than the non-producing strain regardless of filament size and that (ii) longer filaments of the non-saxitoxin-producing strain would be consumed less than shorter ones.

## Materials and Methods

### Phytoplankton Precultivation

Two Brazilian strains of the cyanobacterium *C. raciborskii* were used in this study: LETC CYRF-01, a saxitoxin producer (here after STX+) (≈5 μg saxitoxins g^−1^ dry weight) [27] and LETC CS1, with no detectable saxitoxin and cylindrospermopsin production (here after STX−) [28]. Stock cultures of these strains were maintained in modified WC medium [29] at 25 °C, 60 rpm. Initial algal concentration was ≈5 × 10^6^ mm^3^ mL^−1^. The light cycle was programmed to gradually increase in light intensity to a maximum of 50 μmol photons m^−2^ s^−1^, in a photoperiod of 14 h, in order to mimic the variations of light conditions in nature.

### Precultivation of *C. raciborskii*: Morphology and Cyanotoxin Analysis

Based on the information of the inverse relationship between temperature and *C. raciborskii* filament length [24, 27], we maintained single stock cultures of STX+ and STX− at three different temperatures (17, 25, and 32 °C) for 2 months to obtain cultures that differ in filament length. We expected a median filament length of around 120 μm in 17 °C (long), 90 μm in 25 °C (medium), and 60 μm in 32 °C (short) [27]. Cultures were kept in separate incubators (Sanyo Gallenkamp Orbital Incubator, Loughborough, United Kingdom) at different temperatures and were maintained under the same conditions as stock cultures. All cultures were renewed weekly (7–8 days) with fresh medium to maintain exponential growth, under consideration of previous data on growth curves of both strains at the three different temperatures. After a 2-month incubation period, the length and width of 50 filaments of each culture were measured with a NIKON optical microscope.

Cyanotoxins were measured in samples of *C. raciborskii* cultures harvested during the exponential growth phase. Triplicate samples were freeze-dried on the days of the grazing experiments. Samples of STX+ and STX− strains cultured at the three different temperatures were analyzed for four saxitoxin variants (saxitoxin—STX, neosaxitoxin—NEO, decarbamoylsaxitoxin—dcSTX, and decarbamoylneosaxitoxin—dcNEO) and six gonyautoxins (GTX1-4, decarbomoyl gonyautoxin dcGTX2-3). For this, 40 to 100 mL of a given culture was filtered using a glass fiber filter (Whatman GF/C) and stored in the freezer at −20 °C. Before extraction, samples were lyophilized for 2 h. Toxins were then extracted three times per sample for 10 min at 95 °C in 2.5 mL, 0.1-M hydrochloric acid (HCl) and dried in a Speedvac (Thermo Scientific Savant SPD121P, USA). All water used in this study was purified with a Q-Pod (Millipore, Billerica, USA) and all solvents used were at least of analytical grade. Dried samples were reconstituted in 167-μL water with 0.1 % formic acid (FA), after which 333-μL acetonitrile with 0.1 % FA were added. Reconstituted samples were cleaned by solid-phase extraction (ZIC-HILIC SPE, 500 mg, 3 mL, Merck Sequant AB, Umeå, Sweden): columns were conditioned by adding 3-mL 10 % acetonitrile–90 % water–0.1 % FA and 3-mL 95 % acetonitrile–5 % water–0.1 % FA, washed with 1.5-mL 95 % acetonitrile–5 % water–0.1 % FA, and eluted with 7.5-mL 10 % acetonitrile–90 % water–0.1 % FA. Eluted samples were dried in a Speedvac and reconstituted in 800-μL 20-mM HCl.

Samples were analyzed on an Agilent 1200 LC and an Agilent G6410A QQQ. Samples were separated on an Agilent Zorbax Eclipse XDB-C18 (Santa Clara, CA, USA) 4.6 × 150 mm, 5 μm column by Millipore water with 0.1 % heptafluorobutyric acid (*v*/*v*, eluent A), and acetonitrile with 0.1 % heptafluorobutyric acid (*v*/*v*). Flow rate was 0.4 mL min^−1^, injection volume 5 μL, and column temperature 20 °C. The following gradient was applied: 0 min 5 % B, 2 min 10 % B, 3 min 20 % B, 6 min 20 % B, 10 min 50 % B, 18 min 90 % B with a 9-min postrun at 5 % B and linear increases in B between the time steps. Compounds were detected as shown in Table 1. Capillary voltage was 3000 V. Each compound was identified by two transitions. For identification, the ratio between the quantifier and the qualifier ion had to be within a 20 % relative range of the expected value.

**Table 1:** MS/MS settings for the analyzed compounds

Toxin	Precursor Mass-to-charge ratio	Fragmentor (V)	Quantifier mass-to-charge ratio	Collision energy (V)	Qualifier mass-to-charge ratio	Collision energy (V)
dcGTX2	273.0	110	255.0	10	126.0	20
dcGTX3	353.0	90	255.0	20	335.0	5
GTX1	332.0	120	314.0	30	236.0	30
GTX2	316.0	140	148.0	25	298.0	15
GTX3	396.0	80	298.0	10	378.0	3
GTX4	412.0	90	314.0	20	332.0	10
dcNEO	273.0	92	225.1	20	126.0	20
dcSTX	257.0	92	126.0	20	239.1	12
NEO	316.0	99	298.1	16	220.1	20
STX	300.0	99	204.0	24	282.1	16

Calibration standards for all analyzed toxins were obtained from the National Research Council (Canada). Samples were quantified against an external concentration curve in 20 mM HCl and subsequently corrected for recovery. Recovery was determined by spiking a green alga (*Scenedesmus obliquus* SAG 276/3a) before extraction. If needed, samples were diluted in 20 mM HCl before reanalysis. Recovery ranged from 31 % for GTX2 to 60 % for dcSTX. Compounds with similar mass-to-charge ratios (dcGTX2 and dcNEO; GTX2 and NEO) were well separated in time (retention time difference > 4.5 min). Limit of detection (defined as signal-to-noise >3 for both transitions) ranged from 0.11 pmol per injection for dcSTX to 0.91 pmol per injection for GTX1. Chromatograms are provided in the supplementary material.

### Copepods Sampling and Culture

The calanoid copepod *E. gracilis* was sampled in Lake Rauwbraken (Berkel-Enschot, the Netherlands). This lake has experienced dense cyanobacterial blooms, with high contributions of the species *Planktothrix rubescens*, *Microcystis aeruginosa*, *Aphanizomenon flos-aquae*, *Anabaena* spp., and *Woronichinia naegeliana. P. rubescens* was registered in the lake year-round, while the other species were reported mostly during the summer months. A restoration process was performed in the lake in 2008 after which phosphorus concentration and cyanobacterial biomass drastically decreased [30]. The average chlorophyll *a* concentration in the 2 years prior to restoration was 19.5 (±36.5) μg L^−1^, and dropped to concentrations as low as 3.7 (±4.5) L^−1^ in the years following the treatment, including the period when animals were collected. *C. raciborskii* was never detected in this lake (F. Van Oosterhout, personal communication). Copepods were sampled with a 55-μm plankton net and transported under ambient temperature to the laboratory within 2 h of sampling. The animals were isolated under a dissecting microscope and rinsed three times with distilled water before being transferred to a glass beaker filled with synthetic water similar to surface water in the Netherlands [12]. They were acclimated under laboratory conditions for 5 days before the start of experiments at 22 °C under gentle aeration and fed with the cryptophycean *Cryptomonas pyrenoidifera* (NIVA 2/81), at a rate of 0.5 mg C L^−1^ d^−1^. *C. pyrenoidifera* was cultured in a chemostat with modified WC medium [29] at 25 °C and a light intensity of 50 μmol photons m^−2^ s^−1^ under a photoperiod of 12:12 h (light/dark).

### Grazing Test

We carried out grazing experiments to compare the effect of two different *C. raciborskii* strains (STX+ or STX−) of each with three filament sizes (short, medium, long) on the clearance rates of *E. gracilis*. This was assessed by incubating 2–3 animals for 2–3 h in the dark in 2.5-mL food suspensions in 24-well culture plates at 22 °C (±1). The total food concentration offered to the copepods was equivalent to 0.5 mg C L^−1^. The carbon content of food suspensions was estimated from the biovolume, using a conversion formula (C = aV^b^, where a = 0.1204; b = 1.051; V = biovolume) [31]. The different food treatments were as follows:

1. 100 % STX− short filaments
2. 100 % STX− medium filaments
3. 100 % STX− long filaments
4. 100 % STX+ short filaments
5. 100 % STX+ medium filaments
6. 100 % STX+ long filaments

Each food treatment was replicated four times. All grazing tests were performed in the same week. Copepod clearance rates (CR, in mL ind^−1^ h^−1^) were estimated by the difference in chlorophyll *a* concentrations between wells that contained copepods and wells that did not (controls), determined by PHYTO-PAM [32] using the formula CR = {ln(Chla_control_ – Chla_treatment_)} / ∆t × V/N, in which Chla_control_ is the final algal concentration in the controls, Chla_treatment_ is the final algal concentration in the treatments, ∆t is the incubation time (h), V is the culture volume (mL), and N is the number of animals.

The ingestion rates (IR in mg C ind^−1^ h^−1^) were calculated from CR using the equation

$$ \mathrm{I}\mathrm{R} = \mathrm{C}\mathrm{R}\times \sqrt{A_0\cdot {A}_t} $$

Where $\sqrt{A_0\cdot {A}_t}$ is the geometric mean of the algal concentration during time *t*, A_0_ is the initial algal concentration (mg C L^−1^), and A_t_ is the algal concentration at the end of the experiment (mg C L^−1^).

Copepods were added to food suspensions after 24 h of starvation to minimize differences in the grazing response due to gut fullness. In order to minimize age-related differences, only C5 copepodites and adults were used for the grazing experiments. During the experiments, food suspensions were gently bubbled every hour to prevent settling and copepods were checked for motility. All the animals were perfectly well after the experiments and were removed at the end of the grazing period to avoid interference with fluorescence readings [12].

The clearance rates were compared using two-way ANOVA with strain and filament size as fixed factors. Differences between means were distinguished by the Holm–Sidak post hoc comparison test. Linear regression was used to test whether IR was related to toxin content of the STX+ strain. Statistical analysis was performed with SigmaPlot, version 13.0.

## Results

### *C. raciborskii* Morphology

Filaments of either strain cultivated at 17, 25, and 32 °C varied only slightly in width, whereas temperature strongly affected filament length (Table 2). Filaments of STX+ and STX− strains incubated at 17 °C were the longest (158 and 130 μm, respectively). Filaments from strains incubated at 25 and 32 °C were significantly shorter than the filaments in cultures reared at 17 °C (35-40 % and 65-70 %, respectively) (Table 2). Based on average filament length, we defined the following size classes: (i) short, (ii) medium, and (iii) long (Table 2).

**Table 2:** Average (standard deviation) filament length and width of *C. raciborskii* (STX– and STX+ strains) cultured at different temperatures and size classes of filament length adopted in this study (*N* = 50)

*C. raciborskii* strain	Cultivation temperature	Filament width (μm)	Filament length (μm)	Range filament length (μm)	Size class
STX−	17 °C	2.2 (0.3)	129.9 (32.9)	78.0–237.9	Long
	25 °C	2.1 (0.4)	78.1 (42.1)	35.1–156.0	Medium
	32 °C	2.4 (0.6)	39.1 (19.56)	7.9–99.45	Short
STX+	17 °C	1.9 (0.4)	158.4 (66.6)	87.8–429.0	Long
	25 °C	2.0 (0.3)	102.7 (49.4)	46.9–237.9	Medium
	32 °C	2.2 (0.5)	54.8 (24.7)	17.6–107.3	Short

### *C. raciborskii* Toxins

**Fig. 1:**
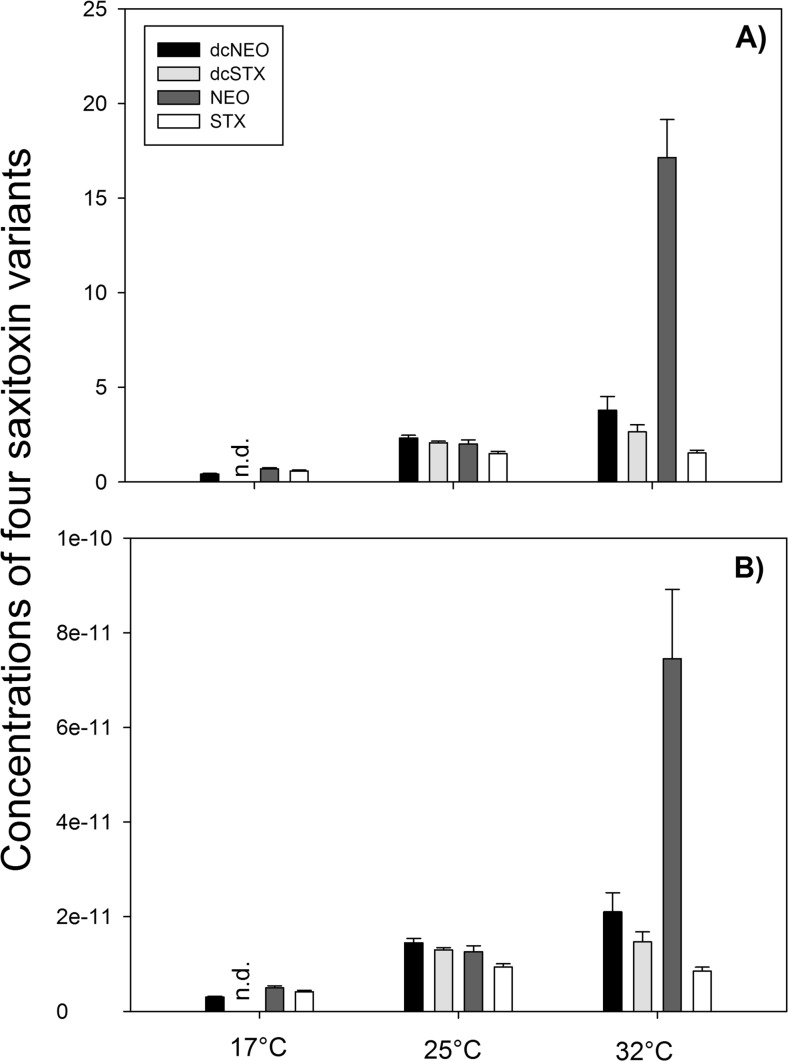
Concentrations of four saxitoxin variants in **a** incubated cultures (μg/L) and **b** per cyanobacteria biovolume (μg/μm^3^) in *Cylindrospermopsis raciborskii* STX + strain grown at different temperatures. *N.d.* means not detected

We did not find any gonyatoxins or saxitoxins in the STX− cultures. In the STX+ cultures, STX, NEO, dcSTX, and dcNEO were detected (Fig. 1). Total saxitoxin concentration (i.e., the sum of the four measured variants) increased at higher temperatures in the STX+ cultures, with 1.7 μg eq STX L^−1^ in cultures incubated at 17 °C, 7.9 μg eq STX L^−1^ at 25 °C, and 25.1 μg eq STX L^−1^ at 32 °C (Fig. 1a). This increase in saxitoxin was not merely a result of higher *C. raciborskii* densities, since the amount of toxins per unit of biovolume increased as well (Fig. 1b). In addition, we observed a difference in the composition of the variants (Fig. 1). The variant dcSTX was not detected in the 17 °C culture but appeared in cultures reared at 25 and 32 °C, while the concentration of NEO tended to increase more than that of the other variants at 25 and 32 °C (Fig. 1).

### Grazing responses to different *C. raciborskii* morphotypes and chemotypes

**Fig. 2:**
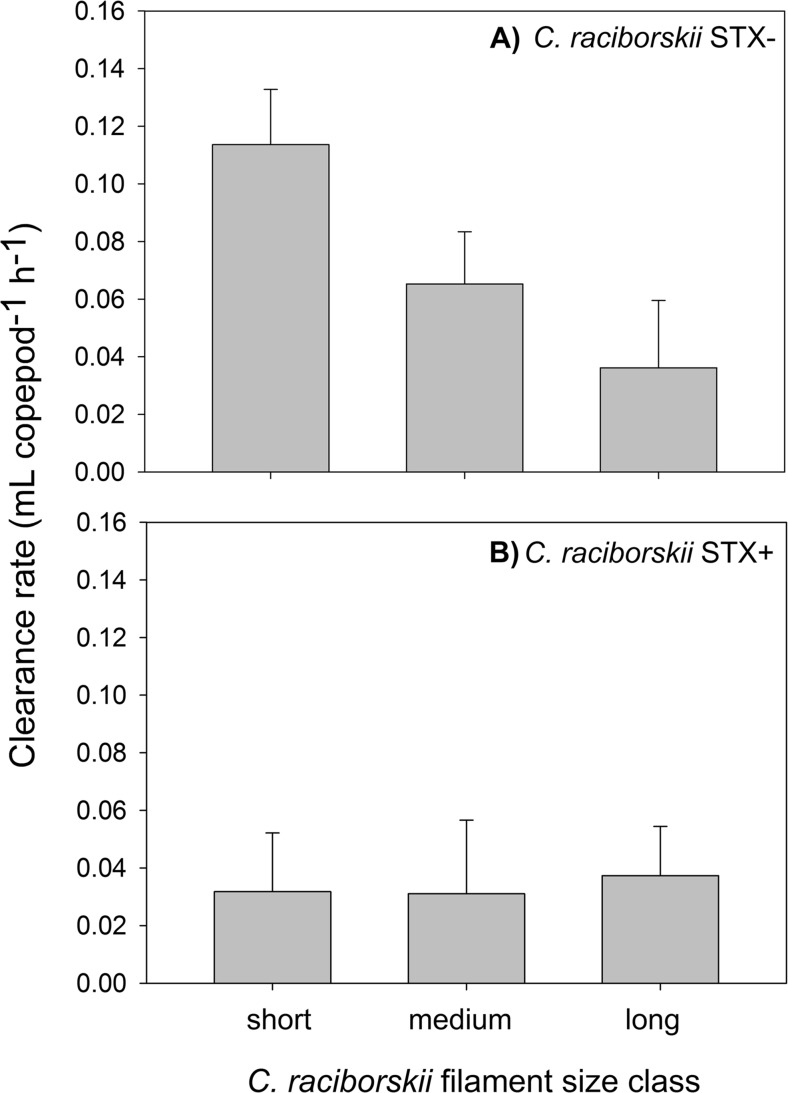
*Eudiaptomus gracilis* clearance rates (CR, mL ind^−1^ h^−1^) feeding on 0.5 mg C L^−1^ of *Cylindrospermopsis raciborskii* STX− (**a**) and STX− (**b**) strains and size class (short, medium, long). (*N* = 4)

**Fig. 3:**
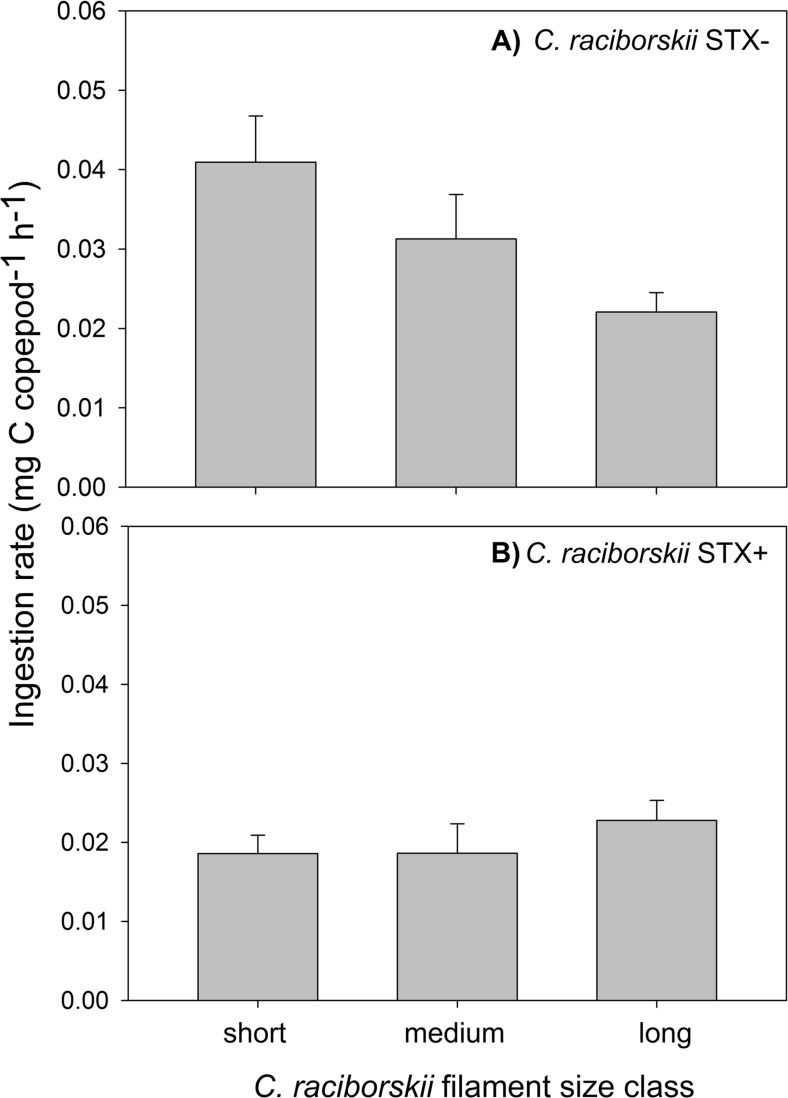
*Eudiaptomus gracilis* ingestion rates (IR, mg C ind^−1^ h^−1^) feeding on 0.5 mg C L^−1^ of *Cylindrospermopsis raciborskii* STX− (**a**) and STX− (**b**) strains and size class (short, medium, long). (*N* = 4)

*E. gracilis* ingested *C. raciborskii* in all treatments, reducing filaments in treatments compared to the controls (Table 3). Clearance rates (CRs) and ingestion rates (IRs) differed significantly among treatments (Figs. 2 and 3). In general, CR of the STX− strain was significantly higher than of the STX+ strain (two-way ANOVA, F_1,23_ = 24.37; *p* < 0.001), suggesting a strong negative effect of saxitoxin on the feeding of *E. gracilis*. An effect of filament size was also observed (two-way ANOVA, F_2, 23_ = 8.92; *p* = 0.002): the Holm–Sidak post hoc comparison test revealed that for the STX− strain, the CR of the small filament culture was significantly higher than that of the larger filament cultures (medium and long). The CR of the smallest size class of STX− filaments was more than twice as high as the CR of the largest size class (long filaments) (Fig. 2a). There was a significant strain × size interaction (two-way ANOVA, F_2, 23_ = 23.27; *p* < 0.001), confirming that the relationship between CR and filament size differed between strains.

**Table 3:** Average *C. raciborskii* filament concentration (STX− and STX+ strains) in the beginning (initial) and at the end of the incubation period; with copepods (copepods) and without copepods (control)

*C. raciborskii* strain	Filament class	Initial (filaments/mL)	Copepods (filaments/mL)	Control (filaments/mL)
STX−	Short	3.0 × 10^4^	2.3 × 10^4^	2.8 × 10^4^
	Medium	1.9 × 10^4^	1.4 × 10^4^	1.6 × 10^4^
	Long	1.0 × 10^4^	6.5 × 10^3^	6.7 × 10^3^
STX+	Short	2.0 × 10^4^	1.9 × 10^4^	1.8 × 10^4^
	Medium	1.1 × 10^4^	1.0 × 10^4^	1.0 × 10^4^
	Long	7.0 × 10^3^	5.8 × 10^3^	6.0 × 10^3^

Overall, IR followed the same pattern as CR (Fig. 3), with significant effects of strain (two-way ANOVA, F_1, 23_ = 22.7; *p* < 0.001), filament size (two-way ANOVA, F_2, 23_ = 15.8; *p* < 0.001), and their interaction (two-way ANOVA, F_2, 23_ = 11.5; *p* < 0.001). However, the Holm–Sidak post hoc comparison test indicated significant differences in IR among all three filament sizes in the STX− strain (Fig. 3).

In spite of the strong feeding inhibition observed in treatments with the STX+ strain, IR was not significantly related to toxin concentration per biovolume (STX eq/ μm^3^; r^2^ adj = 0.07; *F* = 0,05; *p* = 0.814), thus indicating that feeding was inhibited regardless of STX concentration.

## Discussion

### Relationship Between Temperature and Phenotypic Traits

Cyanobacterial blooms are expected to become more abundant and intense with global climate change, promoted by altered temperatures and precipitation rates, leading to new hydrologic regimes and nutrient loads in aquatic systems worldwide [1]. Thus, understanding cyanobacterial responses to changing conditions, such as temperature increase, will enhance our ability to predict the occurrence and magnitude of blooms in the future.

The range of filament sizes observed in our study is within the range generally found for natural *C. raciborskii* populations (35–430 μm; [27]). Morphological changes in *C. raciborskii* in response to changing temperatures were observed previously [24, 27, 33]. The inverse relationship between filament size and temperature found in our study has also been reported for other *C. raciborskii* strains: it was consistently detected in the Brazilian strain CYRF (the STX+ strain used in this study) in previous studies [24, 27], as well as in the STX− strain used in this study (CS1), and two other strains isolated from a pond in Taiwan [33]. Morphological variability related to temperature change also occurs in tropical and subtropical systems, where shorter filaments were observed in the warmer months [24, 34]. This appears to be a consistent trend, although more observations are needed.

Besides the morphological variations mentioned above, our results showed that the *C. raciborskii* STX+ strain used in this study produced more saxitoxins per unit biomass at higher temperatures. Although *C. raciborskii* is expected to further expand its distribution and proliferate with global climate change [18], few studies have investigated the effect of temperature on the production of paralytic shellfish toxins (PSTs) by C*. raciborskii*. In the *C. raciborskii* strain C10, total saxitoxin concentrations were similar in cultures grown at 19 and 25 °C [35]. In contrast, another *C. raciborskii* strain showed a negative relationship between cylindrospermopsin production and temperature, with cylindrospermopsin concentrations dropping below the detection level at 35 °C [36]. To our knowledge, our study is the first to report elevated saxitoxin production in *C. raciborskii* under high temperatures. In addition, we found that NEO increased at a higher rate with increasing temperatures than dcNEO, dcSTX, and STX.

### Grazing Effects

*E. gracilis* reduced grazing on all three filament sizes of the STX+ strain compared to the shortest filaments of the STX− strains. Hence, filament size had no effect on copepod grazing of the STX+ strain. This suggests that *E. gracilis* used chemical cues emitted by the STX+ strain to avoid ingestion of possibly harmful food. However, it is unclear whether these cues are saxitoxins or other chemical compounds. A similar response in a different *C. raciborskii* strain was reported: copepods preferred non-cylindrospermopsin-producing strains to cylindrospermopsin-producing strains, provided that filament lengths were similar [25]. Such feeding responses induced by chemical cues of *C. raciborskii* in copepods are still poorly understood. However, calanoid copepods have the ability to recognize and reject other species of cyanobacteria that produce known cyanotoxins [12, 37]. We also cannot discard the idea that the observed variations in the toxic profiles of cultures of the STX+ strain (different proportions and concentrations of STX, NEO, dcSTX, and dcNEO) may have influenced the feeding behavior of copepods. Most previous studies consider only generalized effects of STX, disregarding the effects of the different variants on zooplankton. The toxic effects of STX and its derivates are generally related to their action on site 1 of the a-subunit of voltage-gated sodium, which promotes a progressive loss of neuromuscular function channels [38]. Furthermore, there may be differences in the toxicity of variants, with STX being the most toxic followed by NEO and GTX 1–3 [38]. Considering this, the possibility remains that the increased toxicity of the STX+ strain, due to higher NEO concentrations, precluded a preference for smaller filaments, similar to that observed for the STX− strain. However, the similarity across STX+ treatments observed in both CR and IR, as well as the lack of a positive relationship among IR and the toxin content/biovolume ratio indicate that the animals are somehow capable of detecting harmful characteristics of this strain prior to ingestion, regardless of toxin concentration. Avoidance of toxic food is an important strategy that reduces susceptibility of grazers to toxic cyanobacteria [39]. Therefore, such cue-based feeding behavior might explain the observed dominance of copepods in environments with toxic cyanobacteria blooms [40].

Our results indicate that the variability in morphology also has a direct effect on the extent of grazing of *C. raciborskii*. Longer filament size significantly reduced the intake of *C. raciborskii* by the copepod *E. gracilis*, but only for the STX− strain. Size is a key trait that affects many functional aspects of phytoplankton, including vulnerability to grazing [41]. Since prey morphology is directly related to zooplankton ingestion efficiency, large filaments and colony sizes may increase the prey’s grazing resistance [42]. However, the consequence of low manageability caused by large prey size is directly related to the feeding mode of grazers [43]. While the feeding appendages of generalist filter-feeding zooplankton may become clogged by the ingestion of particles of inadequate size [44], selective grazers, like some copepods, are able to handle and cut the cyanobacteria filaments to an edible size [13, 45]. However, the requirements of dealing with particularly long prey may increase handling time and costs and therefore reduce ingestion efficiency [39]. The optimum prey size of filter feeding pelagic copepods is thought to be 2–5 % of their prosoma length [46]. Based on this relationship, the estimated optimum prey size for *E. gracilis* ranges from about 30–80 μm, roughly corresponding to the smallest filament size (average 39.1 and 54.8 μm in STX− and STX+, respectively) in our experiment. This is also consistent with the optimum range of prey size, reported to be between 10 and 50 μm for diaptomids [26]. Given that grazing on the STX− strain of *C. raciborskii* decreased with larger filament sizes, our results suggest that food particles outside the optimum size range are less exposed to grazer pressure. Overall, since size effects were not observed for the SXT+ strain, morphology may have a secondary effect on grazing that is only relevant in the absence of specific chemical cues.

Our data suggest that *C. raciborskii*’s response to grazing may be related to temperature fluctuations, which may produce different morphotypes and chemotypes in nature. This implies that seasonal and regional variations in temperature as well as the predicted future climate change will likely produce multiple ecotypes, with different vulnerability to grazing. Thus, the contrasting feeding responses of *E. gracilis* to the cultures offered in this study indicate that the phenotypic plasticity of *C. raciborskii* might determine its success when dealing with grazing pressure. In general, grazing of toxin-producing strains is likely to be low, especially by selective zooplankton, which is capable of recognizing the chemical cues that are abundant in bloom-dominated systems. This may be a competitive advantage to the toxigenic strains, which could facilitate bloom proliferation and consequently increase the risk of toxic blooms. While toxic strains appear to be more resistant to selective grazers, size might be relevant in non-toxic strains. In the light of the predicted global warming, such responses to temperature will most likely alter the susceptibility of *C. raciborskii* to grazing, leading to reduced zooplankton grazing and higher biomass, and even more importantly, to considerably higher toxin concentrations, although more studies are needed to confirm this.

Our findings indicate important mechanisms by which cyanobacteria reduce grazing losses. However, the controlled conditions and single diets used in this study are still far from the conditions that copepods experience in the field. Under natural conditions, even during blooms, other food sources are available, such as different types of non-toxic phytoplankton and heterotrophic microbes. Yet, the efficiency of copepod selectivity, including that of *E. gracilis* [12], tends to increase with the availability of other nutritious food sources [25]. Hence, the use of mixed diets with alternative food sources is not expected to change the current conclusions (based on single food diets) regarding the relative role of toxicity versus morphological traits in the feeding of selective grazers. Based on our results from single diets, the observed cue-based, strong avoidance of cyanobacteria by copepods would be even more drastic under natural conditions when alternative good food sources are present.

The *C. raciborskii* cultures maintained at different temperatures could also have differed in aspects other than morphology and saxitoxin production, as temperature may influence the biochemical makeup of phytoplankton [47]. Therefore, relevant differences in food quality (i.e., fatty acid composition) induced by temperature also might have affected food selection by copepods in this study [48]. However, a recent study has shown that taxonomic affiliation accounts for more variation in the composition of fatty acids in phytoplankton than the most important variables influencing growth conditions, including temperature [47]. Thus, although we do not have such information for the cultures used in this study, they are likely to be more similar to each other than to other strains or species in relation to cell biochemistry.

We also have to consider that the grazing responses observed in this study could have been very different to the responses of zooplankton populations with varying levels of previous exposure to toxic bloom-forming cyanobacteria. Zooplankton from Lake Rauwbraken was constantly exposed to the cyanobacteria *Anabaena* spp., *A. flos-aquae*, *M. aeruginosa*, *P. rubescens*, and *W. naegeliana* before its restoration in 2008. Phytoplankton biomass has been low ever since, including the sampling period for copepods used in this study. Therefore, we cannot exclude the idea that this previous exposure to diverse cyanobacterial toxic compounds and morphologies might have enhanced zooplankton tolerance and selectivity. Considering that such a response may be common in natural environments, this also highlights the different potential of copepod grazers to adapt to harmful cyanobacteria.

In conclusion, by separating the effects of morphology (i.e., filament length) and toxicity (i.e., saxitoxin content) against selective grazers, we provide evidence of their distinct protective function for an invasive and globally spreading bloom-forming cyanobacterium that allows it to resist ingestion by commonly occurring zooplankton. More importantly, we observed a trend of chemical traits to overrule the effect of morphological features in *C. raciborskii*, as the toxic strains appear to be more capable of resisting selective grazers, regardless of their size. On the other hand, non-toxic strains appear to be less vulnerable to grazing if they are larger. Overall, results indicate that cyanobacterial toxicity may be the primary line of defense against grazers, though morphology plays a critical role in the absence of toxins. The great plasticity of morphological and chemical traits of *C. raciborskii* and the consequent contrasting effects they pose on the feeding behavior of selective zooplankton might explain the success of this cyanobacterium in a variety of aquatic environments.

## Electronic supplementary material

Below is the link to the electronic supplementary material.

ESM 1 (GIF 109 kb)

High Resolution Image (TIF 2068 kb)
