# Supplementary figures and images for: Toxicity Overrides Morphology on Cylindrospermopsis raciborskii Grazing Resistance to the Calanoid Copepod Eudiaptomus gracilis
Source: Microb Ecol. 2016 Feb 18;71:835–44. doi: 10.1007/s00248-016-0734-8 (PMC4823325; doi:10.1007/s00248-016-0734-8)

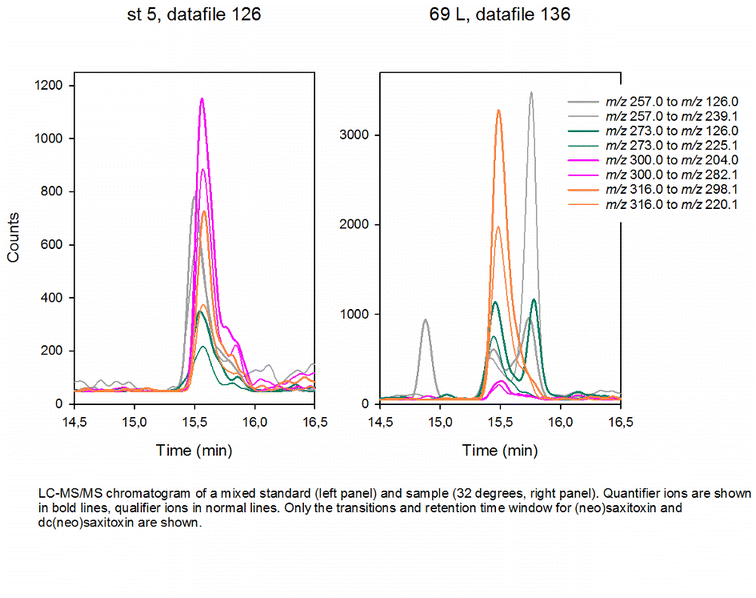

Supplement: Supplementary file 1 — (GIF 109 kb) [file 248_2016_734_Fig5_ESM.gif]

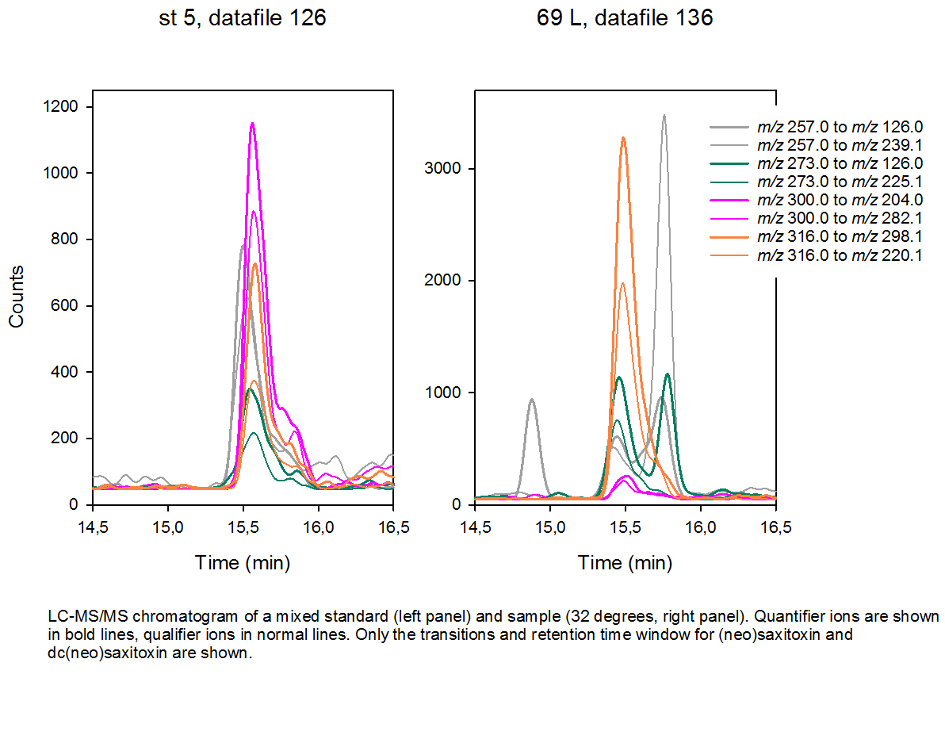

Supplement: Supplementary file 2 — High Resolution Image (TIF 2068 kb) [file 248_2016_734_MOESM1_ESM.tif]
